# The combination of propylene glycol and vegetable glycerin e-cigarette aerosols induces airway inflammation and mucus hyperconcentration

**DOI:** 10.1038/s41598-024-52317-8

**Published:** 2024-01-23

**Authors:** Michael D. Kim, Samuel Chung, Nathalie Baumlin, Jian Qian, Robert N. Montgomery, Juan Sabater, Cory Berkland, Matthias Salathe

**Affiliations:** 1 Department of Internal Medicine, Division of Pulmonary, Critical Care and Sleep Medicine, University of Kansas Medical Center, Kansas City, KS 66160 USA; 2 Department of Pharmaceutical Chemistry, University of Kansas, Lawrence, KS 66047 USA; 3 Department of Biostatistics and Data Science, University of Kansas Medical Center, Kansas City, KS 66160 USA; 4 Department of Research, Mount Sinai Medical Center, Miami Beach, FL 33140 USA

**Keywords:** Mechanisms of disease, Chronic obstructive pulmonary disease, Ion channels

## Abstract

Despite concerns over their safety, e-cigarettes (e-cigs) remain a popular tobacco product. Although nicotine and flavors found in e-cig liquids (e-liquids) can cause harm in the airways, whether the delivery vehicles propylene glycol (PG) and vegetable glycerin (VG) are innocuous when inhaled remains unclear. Here, we investigated the effects of e-cig aerosols generated from e-liquid containing only PG/VG on airway inflammation and mucociliary function in primary human bronchial epithelial cells (HBEC) and sheep. Primary HBEC were cultured at the air–liquid interface (ALI) and exposed to e-cig aerosols of 50%/50% v/v PG/VG. Ion channel conductance, ciliary beat frequency, and the expression of inflammatory markers, cell type-specific markers, and the major mucins MUC5AC and MUC5B were evaluated after seven days of exposure. Sheep were exposed to e-cig aerosols of PG/VG for five days and mucus concentration and matrix metalloproteinase-9 (MMP-9) activity were measured from airway secretions. Seven-day exposure of HBEC to e-cig aerosols of PG/VG caused a significant reduction in the activities of apical ion channels important for mucus hydration, including the cystic fibrosis transmembrane conductance regulator (CFTR) and large conductance, Ca^2+^-activated, and voltage-dependent K^+^ (BK) channels. PG/VG aerosols significantly increased the mRNA expression of the inflammatory markers interleukin-6 (*IL6*), *IL8*, and *MMP9*, as well as *MUC5AC*. The increase in *MUC5AC* mRNA expression correlated with increased immunostaining of MUC5AC protein in PG/VG-exposed HBEC. On the other hand, PG/VG aerosols reduced MUC5B expression leading overall to higher MUC5AC/MUC5B ratios in exposed HBEC. Other cell type-specific markers, including forkhead box protein J1 (*FOXJ1*), keratin 5 (*KRT5*), and secretoglobin family 1A member 1 (*SCGB1A1*) mRNAs, as well as overall ciliation, were significantly reduced by PG/VG exposure. Finally, PG/VG aerosols increased MMP-9 activity and caused mucus hyperconcentration in sheep in vivo. E-cig aerosols of PG/VG induce airway inflammation, increase MUC5AC expression, and cause dysfunction of ion channels important for mucus hydration in HBEC in vitro. Furthermore, PG/VG aerosols increase MMP-9 activity and mucus concentration in sheep in vivo. Collectively, these data show that e-cig aerosols containing PG/VG are likely to be harmful in the airways.

## Introduction

The use of e-cigarettes (e-cigs) continues to increase worldwide despite concerns over their safety. There were an estimated 68 million e-cig users (or vapers) globally in 2020, increasing from a total of 58.1 million vapers in 2018^1^. Especially concerning is the use of e-cigs among adolescents, with “ever-use” of e-cigs exceeding 30% of teenagers in North America and Europe between 2018–2019^2,3^. This is particularly alarming given reports that adolescent vapers are at increased risk of chronic bronchitis symptoms compared to adolescents who never used e-cigs^4,5^. Thus, understanding how e-cig aerosols impact the airways remains critically important in determining the long-term health risks of these commonly used tobacco products.

E-cig liquids (e-liquids) typically contain nicotine and flavorings with the common delivery vehicles propylene glycol (PG) and vegetable glycerin (VG) comprising the bulk of the formulation. Many flavored e-liquids as well as flavoring chemicals used in e-liquids have been shown to be cytotoxic^6–9^, and the negative effects of nicotine-containing e-liquids in the lungs of animal models have been well documented by us and others^10–14^. The airway epithelium provides the first line of defense against inhaled substances and the ability of the airway to clear mucus is critical to prevent obstruction and stave off infection^15^. E-cig aerosols have further been shown to disrupt many of the processes important for normal mucociliary clearance, including ciliary beating, mucus production, and the activities of ion channels required for proper airway hydration^10,11,16–18^. Although many of these effects have been attributed to nicotine and possible synergistic effects of nicotine with flavorings, PG and VG aerosols may also contribute to the mucociliary dysfunction induced by e-cigs.

We recently showed that exposure of primary human bronchial epithelial cells (HBEC) to e-cig aerosols of 100% VG, in the absence of any nicotine or flavors, reduced the function of cystic fibrosis transmembrane conductance regulator (CFTR), an anion channel important for mucus hydration^19^. HBEC exposed to VG aerosols further showed reduced ciliary beating, mucus hyperconcentration, and increased expression of inflammatory cytokines^19^. On the other hand, e-cig aerosols of 100% PG reduced the activity of the large conductance, Ca^2+^-activated, and voltage-dependent K^+^ (BK) channel in exposed HBEC, contributing to mucociliary dysfunction^20^. Interestingly, sheep exposed to e-cig aerosols of 100% PG or 100% VG exhibited mucus hyperconcentration and elevated activity of matrix metalloproteinase-9 (MMP-9) measured from airway secretions^19,20^. MMP-9 activity has been previously shown to be elevated in the lungs and upper airways of vapers^13,21^, and MMP-9 is known to play an important role in the pathogenesis of chronic obstructive pulmonary disease (COPD)^22^.

In this study, we set out to determine whether e-cig aerosols containing both PG and VG in equal ratios, which is relevant to real-world e-cig use, affect mucociliary function and inflammation in the airway epithelium. We found that PG/VG aerosols disrupt the normal function of both CFTR and BK channels in primary HBEC in vitro. PG/VG aerosols further reduced ciliary beating and increased the expression of MUC5AC, possibly by inducing goblet cell hyperplasia. PG/VG e-cig aerosols also increased mucus concentration and MMP-9 activity in sheep airways in vivo. Collectively, these data provide further evidence of the harmful effects of PG and VG in the airways.

## Methods

### Study approvals

All experimental procedures and protocols were reviewed and approved by the University of Kansas Medical Center (KUMC) Institutional Biosafety Committee. The KUMC Institutional Review Board determined the consent of organ donation for research covers research use of this material and, since lungs were obtained from deceased individuals with minor, de-identified information, its use is not considered human subjects research as defined by CFR 46.102. All experimental procedures and protocols in sheep were approved and performed in accordance with guidelines from the Mount Sinai Medical Center Animal Research Committee. Reporting of study results are in accordance with ARRIVE guidelines.

### E-cig device and e-liquids

The eVic™ Supreme with a Delta 23 atomizer (Joyetech®, Shenzen, China) was used to generate e-cig aerosols for all experiments as previously described^11,19,20^. The Delta 23 uses the C3 atomizer head which is a 3-coil device with a resistance of 1.4 Ω. The Delta 23 was set to 3.2–3.6 V with a power setting of ~ 7.0 W. 100% PG and 100% VG were made by Archer–Daniels–Midland (Chicago, IL, USA) and dispensed directly into new plastic containers and supplied by the American E-liquid Store (Wauwatosa, WI, USA). The 50%/50% v/v PG/VG blend was specifically mixed using the same pure components under cGMP guidelines by Pace Engineering Concepts (Delafield, WI, USA) to assure quality control. All e-liquids contained no nicotine or flavoring chemicals.

### Particle size distribution of generated e-cig aerosols using a cascade impactor

Aerosol size distributions were analyzed using an eight-stage COPLEY Andersen cascade impactor (ACI; Copley Scientific Limited, Nottingham, UK). ACI experiments were operated at a flow rate of 28.3 L/min for 5 s. Cutoff particle aerodynamic diameters at below flow rate for each stage of the impactor were stage 0 (9.00 µm), stage 1 (5.8 µm), stage 2 (4.7 µm), stage 3 (3.3 µm), stage 4 (2.1 µm), stage 5 (1.1 µm), stage 6 (0.7 µm), stage 7 (0.4 µm), and filter (< 0.4 µm). The aerosol was generated by a Joyetech™ Delta atomizer (set to 3.2–3.6 V with a power setting of ~ 7.0 W) using 100% PG, 100% VG, or 50%/50% v/v PG/VG as the e-liquid. The aerosol from the atomizer was collected by a 60 mL syringe with a collection rate of 10 mL/s for 5 s (about 50 mL aerosol total). Then, the collected aerosol was immediately injected into the ACI within a 4 s duration. The aerosol collection and injection processes were repeated 40 times to ensure measurable mass collection on each collection plate of ACI. The droplets on the collection plates were weighed by a precision balance (the collection plates were pre-weighed before the experiments). Although some condensation of liquid on the inner surface of the syringe was observed, the amount of condensed liquid was estimated to be < 10% of the total amount of liquid collected in the ACI. The amount of adsorbed aerosol was not expected to significantly influence the size and size distribution of the aerosol and was therefore not included in the deposition profile. All ACI experiments were performed at 24 °C in triplicate.

### Air–liquid interface (ALI) cultures

ALI cultures were established from primary human bronchial epithelial cells (HBEC) isolated from the lungs of donors with no known history of smoking, vaping, or airway disease. Lungs were provided by organ procurement agencies, Life Alliance Organ Recovery Agency (University of Miami, Miami, FL, USA), LifeCenter Northwest (Bellevue, Washington, USA), the Nevada Donor Network (Las Vegas, Nevada, USA), and the Midwest Transplant Network (Westwood, Kansas, USA), after being rejected for transplant and with informed consent. Characteristics of donor lungs are shown in Supplementary Table S1. Briefly, HBEC were expanded in Bronchial Epithelial Cell Growth Medium (BEGM) before being seeded on Transwell inserts (#3460; Corning, NY, USA) or Snapwell inserts (#3801; Corning) at a density of 2 × 10^5^ cells in ALI media. ALI media was prepared according to established protocols^23^. The cells were kept submerged in ALI media until reaching confluency, at which point they were exposed to air. The apical surface was washed with Dulbecco’s phosphate-buffered saline (#21-030-CV; Corning) and the ALI media in the basolateral compartment was replaced every other day. Cells were allowed to differentiate on air for ≥ 4 weeks before experiments were conducted.

### E-cig aerosol exposure of HBEC in vitro

The VC 1 Smoking Machine (Vitrocell, Waldkirch, Germany) was used for in vitro exposures of e-cig aerosols as previously described^11,19,20^. A puff of e-cig aerosol was generated from PG/VG e-liquid using the eVic™ Supreme during 3 s of aerosol collection. ALI cultures were exposed to either filtered air or 50 puffs of aerosol (55 mL per puff, applied once every 20 s for 16.7 min). Each puff of e-cig aerosol was diluted with humidified air (relative humidity > 50%) at 0.25 L/min. A constant vacuum was applied at 5 mL/min to generate sufficient flow for e-cig aerosol exposure onto the apical surface of ALI cultures. HBEC were exposed to 50 puffs per session in the morning and evening (100 total puffs per day) for five or seven consecutive days. Basolateral media was changed every other day and the apical surface of ALI cultures was unwashed to preserve the accumulated mucus during the experiments. Immunofluorescence staining and measurements of ion channel conductance, ciliary beat frequency (CBF), and mRNA expression were conducted 4–6 h after the last exposure.

### Determination of e-cig aerosol deposition with a microbalance

To determine the average deposition of PG/VG e-cig aerosols in our in vitro exposure system, aerosol generated as above (50 puffs) was deposited onto a laser-cut 0.2 cm^2^ filter paper, placed on top of a cell-free Transwell filter. After exposure, the added weight was measured on a UMX2 microbalance (Mettler-Toledo, Columbus, OH, USA) and the weight was corrected for fluid density.

### Measurement of ion channel conductance

HBEC were mounted in Ussing chambers connected to a VCC MC6 or MC8 voltage clamp unit (Physiological Instruments, San Diego, CA, USA). CFTR activity was measured as the change in short-circuit current (I_*SC*_) caused by CFTR inhibition with 10 µM CFTR_inh_-172 (#C2992; MilliporeSigma, Burlington, MA, USA) after CFTR stimulation with 10 µM forskolin (#F3197; MilliporeSigma) in the presence of 10 µM amiloride (#A7410; MilliporeSigma) under a basolateral-to-apical Cl^−^ gradient. ENaC activity was measured as the change in I_*SC*_ after amiloride. For measurements of BK channel function, the basolateral membrane of HBEC mounted in Ussing chambers was permeabilized with 20 µM Amphotericin B (#A2411; MilliporeSigma), 10 µM Nigericin (#4312; Bio-Techne Corporation, Minneapolis, MN, USA), and 10 µM Valinomycin (#3373; Bio-Techne Corporation). BK channel activity was then measured as the change in I_*SC*_ following stimulation with 10 µM ATP (#A9187; MilliporeSigma) in the presence of 10 µM amiloride under a basolateral-to-apical K^+^ gradient and an apical-to-basolateral Na^+^ gradient as previously described^24^.

### Analysis of mRNA expression with quantitative real-time PCR (qPCR)

RNA was isolated from HBEC with the E.Z.N.A. Total RNA Kit (OMEGA Bio-tek, Norcross, GA, USA) and cDNA was made with the iScript cDNA synthesis kit (Bio-Rad, Hercules, CA, USA). qPCR was performed using TaqMan™ Gene Expression Assays (Thermo Fisher Scientific, Waltham, MA, USA) for *FOXJ1* (Hs00230964_m1), *IL6* (Hs00985639_m1), *IL8* (Hs00174103_m1), *KRT5* (Hs00361185_m1), *MMP9* (Hs00234579_m1), *MUC5AC* (Hs01365601_m1), *SCGB1A1* (Hs00171092_m1), and *TGFB1* (Hs00998133_m1). *GAPDH* (Hs99999905_m1) was used as endogenous control.

### Immunofluorescence staining

Immunofluorescence staining of HBEC was performed as previously described^20,25^. ALI cultures were fixed with a solution of 50%/50% methanol/acetone for 2 min at – 20 °C followed by three washes with PBS. Fixed cultures were incubated with anti-MUC5AC antibody (MA1-38223; Thermo Fisher Scientific) at 0.4 µg/mL and anti-MUC5B antibody (#37-7400; Thermo Fisher Scientific) at 2 µg/mL overnight at 4 °C. Cultures were then incubated with Alexa Fluor 555 donkey anti-mouse IgG (#A31570; Thermo Fisher Scientific) at 1 µg/mL and Alexa Fluor 488 donkey anti-rabbit IgG (#A21206; Thermo Fisher Scientific) at 1 µg/mL for 1 h at room temperature. Anti-acteylated α-tubulin antibody (#T6793; MilliporeSigma) was used at 1:1000 dilution overnight at 4 °C. Hoechst 33258 (#H3569; Thermo Fisher Scientific) was used at 2 µg/mL for 10 min at room temperature.

### Confocal imaging and image analysis

Z-stack images were obtained using a Nikon C2 + confocal microscope (Nikon Instruments, Tokyo, Japan) with a 20 × objective from six different points on each culture. Quantification of MUC5AC, MUC5B, and Hoechst surface area staining was measured from collapsed z-stack images using ImageJ software (Bethesda, MD, USA). Images were converted to 8-bit grayscale and thresholded using Otsu’s method^26^. The % surface area labeling was measured as the percentage of pixels in the image using the Nucleus Counter plugin for ImageJ.

### Measurement of ciliary beat frequency (CBF)

CBF recordings were measured from four different points within a 3 mm radius from the center of the ALI culture to avoid influence from liquid meniscus. ALI cultures were not washed prior to CBF measurements. CBF was recorded using SAVA software with a Basler acA645 camera (Basler, Ahrensburg, Germany) mounted on a Zeiss Axiovert inverted microscope (Carl Zeiss, Jena, Germany). SAVA software was used to calculate the CBF from whole field analysis (WFA) to avoid selection bias^27^.

### Basolateral addition of PG/VG e-liquid

50%/50% v/v PG/VG at a final concentration of 0.3% or mannitol at a final concentration of 0.74% was added to the basolateral media of ALI cultures of primary HBEC for 24 h before assessing ion channel conductance as described above.

### Animal study design

Adult female sheep (ewes) were used in all experiments because male sheep are naturally more aggressive and thus not amenable for long-term experimentation without the use of general anesthesia. All procedures were in compliance with the Mount Sinai Medical Center Animal Research Committee.

### E-cig aerosol exposure of sheep

Conscious adult ewes were nasally intubated and exposed to aerosols generated from an eVic™ Supreme containing 50%/50% PG/VG e-liquid. Aerosols were drawn into a 60-mL syringe and then delivered into the trachea only during inspiration, at a frequency of 20 breaths/min and a tidal volume of 500 mL, via the inspiration tubing of a piston respirator. Adult ewes were exposed to 80 puffs (40 puffs per session twice daily with ≥ 240 s daily puff time) of PG/VG aerosols for five consecutive days. Tracheal secretions were collected by direct suction^28^ at baseline and at day 5, 1 h after aerosol exposures. Five different sheep were used for these experiments.

### Measurement of mucus concentration (% mucus solids) from sheep tracheal secretions

The percentage of mucus solids in sheep tracheal secretions was measured and calculated as previously described^11,28^. Briefly, the wet weight of mucus in an aluminum weigh boat was measured using a UMX2 microbalance before drying overnight at 65 °C. The dry weight was then measured and wet to dry ratios were calculated as percent solids^29^.

### Measurement of MMP-9 activity from sheep tracheal secretions

MMP-9 activity was measured from sheep tracheal secretions using a Human Active MMP-9 Fluorokine E kit (#F9M00; R&D Systems), validated for use with sheep.

### Statistics

Data were analyzed by PRISM V9 software (GraphPad, San Diego, CA, USA) or using R (R Core Team). All data were analyzed using non-parametric statistics. Linear mixed model (LMM) permutation tests were used to account for technical replicates within the same lung or sheep. Given the small sample sizes and non-normality of the residuals we used LMM permutation tests to evaluate the strength of the associations. For each endpoint, we permuted the exposure (Air vs. PG/VG) or the time indicator (Baseline vs. Day 5) and fit the mixed models with a random intercept for each subject on each permuted data set. The permutation p-value was calculated as the number of permuted coefficients that were as or more extreme than our observed value. The number of permutations, 250,000, was chosen to give a high degree of certainty for the permutation p-value, specifically, a 99% confidence interval for the p-values would have a precision (half width) of approximately 0.001. A key assumption of permutation tests is exchangeability and therefore, we permuted the exposure conditional on the subject (e.g., exposure was permuted on the HBEC values from a single donor, not across donors) and kept the numbers of permuted observations under each condition the same as the observed results. Nevertheless, the assumption of exchangeability is unverifiable. To account for this, we conducted sensitivity analyses where we averaged all responses within each individual subject (donor or sheep) and conducted paired permutation t-tests. Additionally, comparisons of p values for LMM permutation tests vs. paired permutation t-tests are shown in Supplementary Table S2. Sample sizes were not sufficient for sex-specific analyses. Additional information regarding statistical analyses is described in the figure legends.

## Results

### E-cig aerosol characteristics

**Figure 1:**
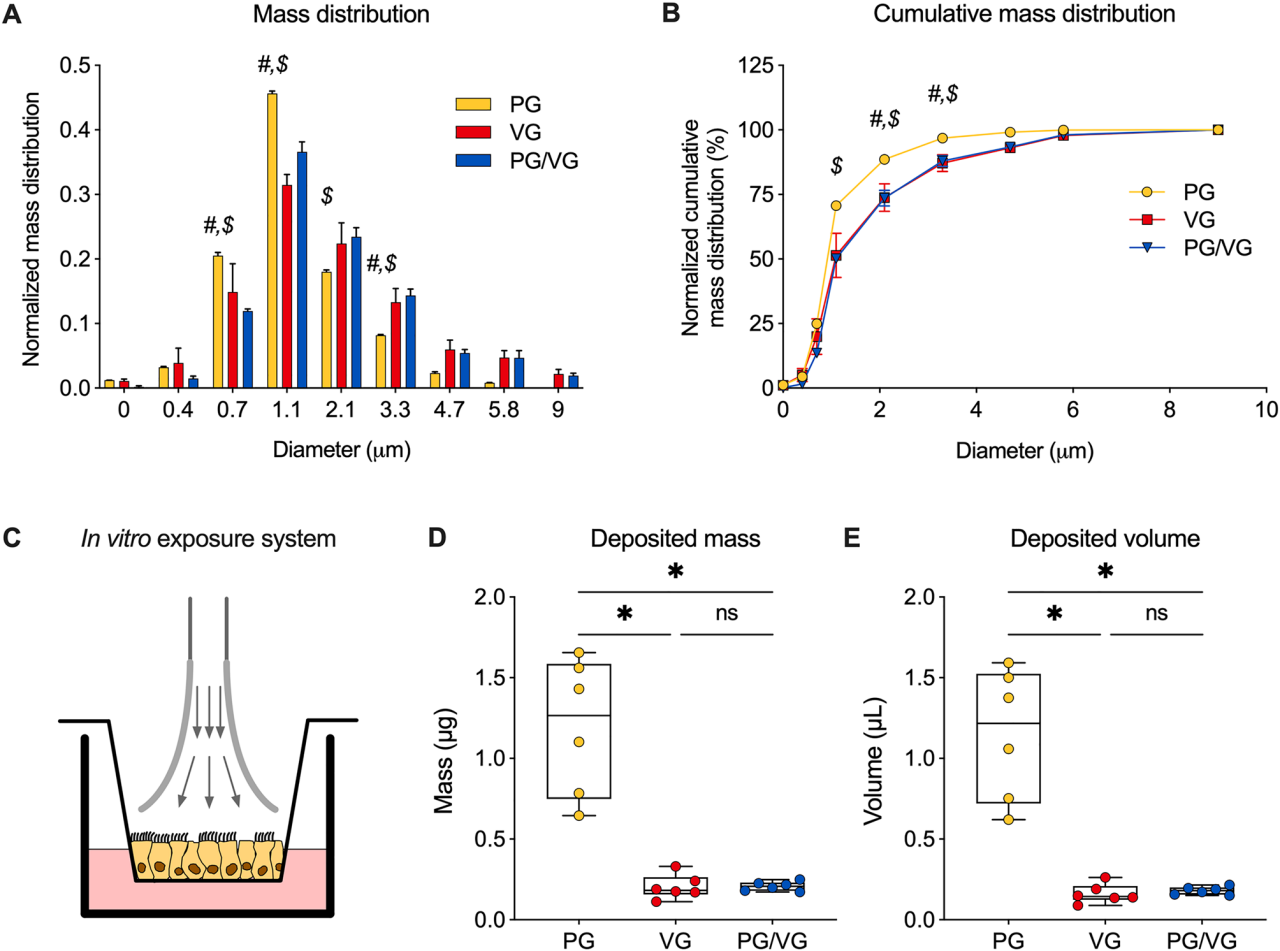
E-cig aerosol characteristics. (**A**) Mass distribution and (**B**) cumulative mass distribution of collected PG, VG, and PG/VG e-cig aerosols using a cascade impactor, suggesting that PG/VG aerosols have similar characteristics to VG aerosols and PG aerosols are smaller than VG and PG/VG aerosols. n = 3. (**C**) Representative schema illustrating exposure of fully differentiated primary HBEC to e-cig aerosols via trumpet inlets from the Vitrocell VC 1. (**D**, **E**) Measurements of aerosol deposition mass (**D**) and volume (**E**) from laser-cut paper put onto a Transwell insert and exposed to PG, VG or PG/VG aerosols via the Vitrocell VC 1 exposure robot (50 puffs). n = 6. *Statistics:* (**A**, **B**) Data were analyzed by two-way ANOVA followed by Tukey post hoc test with *#* indicating p < 0.05 comparing PG vs. VG and *$* indicating p < 0.05 comparing PG vs. PG/VG (**A**, **B**). (**D**, **E**) Data shown as median (line), 25th to 75th percentiles (box), and minimum to maximum values (whiskers). *p < 0.05, Kruskal–Wallis test.

We first characterized the size and mass distribution of e-cig aerosols of 100% PG, 100% VG, and 50%/50% v/v PG/VG to determine whether aerosols created with the same atomizer (Delta atomizer set to 3.2–3.6 V with a power setting of ~ 7.0 W, eVic™ Supreme, Joyetech®, Shenzhen, China) would be similar or meaningfully different among different e-liquids. E-cig aerosols were directly injected into an eight-stage COPLEY Andersen cascade impactor. A greater portion of PG aerosols deposited on the 0.7- and 1.1-µm plates than VG or PG/VG aerosols (Fig. 1A). In contrast, more PG/VG aerosols deposited on the 2.1- and 3.3-µm plates than PG aerosols (Fig. 1A). Cumulative relative mass of PG aerosols deposited on the 1.1-µm plate (70.6%) was significantly greater than PG/VG aerosols (50.2%) (Fig. 1B). This trend extended to the 2.1- and 3.3-µm plates (Fig. 1B). These data suggest that most PG aerosols were smaller in diameter than VG or PG/VG equivalents. The aerosol properties of the PG/VG e-liquid were more closely related to those of VG.

We next investigated the deposition of e-cig aerosols of PG, VG and PG/VG on primary HBEC cultured at the air–liquid interface (ALI), exposed under realistic conditions using the Vitrocell VC 1 and an eVic™ Supreme (Fig. 1C). Mass deposition of aerosols by the VC 1 was assessed using a microbalance and converted to volume to account for the different densities (Fig. 1D,E). VG and PG/VG aerosols did not deposit differently on a surrogate membrane measuring 0.2 cm^2^ in Transwell inserts (Fig. 1D,E). However, we observed significantly higher deposition of PG aerosols compared to VG and PG/VG aerosols, despite identical atomizer settings used for these measurements, but consistent with observed differences using the cascade impactor.

### PG/VG aerosols cause ion channel dysfunction

**Figure 2:**
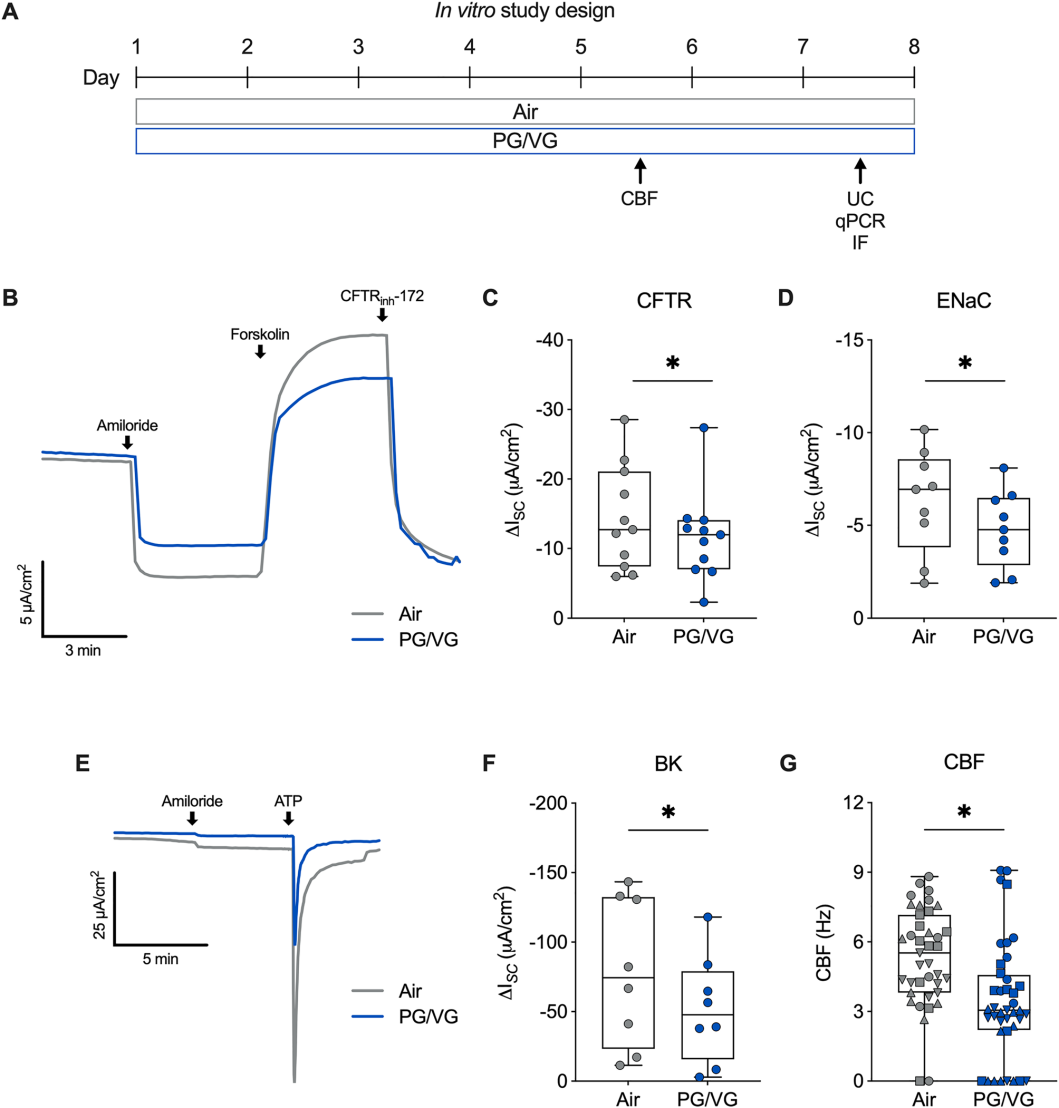
PG/VG aerosols cause ion channel dysfunction. (**A**) Study design for in vitro exposures of primary HBEC to e-cig aerosols of PG/VG. *CBF* ciliary beat frequency, *UC* Ussing chamber, *qPCR* quantitative PCR, *IF* immunofluorescence staining. (**B**) Representative tracing of short-circuit current (I_*SC*_) measurements in Ussing chambers with a basolateral-to-apical Cl^−^ gradient following forskolin stimulation and CFTR inhibition by CFTR_inh_-172 in the presence of amiloride in fully differentiated HBEC exposed to air (control) or e-cig aerosols of PG/VG. (**C**) CFTR conductance as measured by ∆I_*SC*_ following CFTR_inh_-172 in HBEC exposed to air or PG/VG aerosols for seven days. n = 11 ALI cultures from 11 donors. (**D**) ENaC conductance as measured by ∆I_*SC*_ following amiloride in HBEC exposed to air or PG/VG aerosols for seven days. n = 9 ALI cultures from 9 donors. (**E**) Representative tracing of I_*SC*_ measurements in Ussing chambers with a basolateral-to-apical K^+^ gradient following ATP stimulation in the presence of amiloride in fully differentiated HBEC exposed to air or e-cig aerosols of PG/VG. (**F**) BK conductance as measured by ∆I_*SC*_ following ATP in HBEC exposed to air or PG/VG aerosols for seven days. n = 8 ALI cultures from 8 donors. (**G**) Quantification of ciliary beat frequency (CBF) from whole field analysis in HBEC exposed to air or PG/VG aerosols for five days. n = 8 ALI cultures from 4 donors (represented by different shapes), which were sampled 5 times each, resulting in 40 datapoints included in the permutation analysis and shown in the figure. *Statistics:* Data shown as median (line), 25th to 75th percentiles (box), and minimum to maximum values (whiskers). *p < 0.05, Wilcoxon test (**C**, **D** and **F**) or LMM permutation test (**G**).

We recently showed that seven-day exposure of HBEC to e-cig aerosols of 100% VG and 100% PG caused CFTR and BK channel dysfunction, respectively^19,20^. We therefore tested whether e-cig aerosols of 50%/50% v/v PG/VG similarly affect ion channel function. For these experiments, ALI cultures of HBEC were exposed to 100 puffs per day (50 puffs per session twice daily) with a 3 s puff duration (total 300 s daily puff time) of PG/VG aerosols or air (control) for seven consecutive days (Fig. 2A). CFTR, epithelial sodium channels (ENaC), and BK channel conductance were measured in Ussing chambers after seven days. PG/VG aerosols significantly reduced CFTR conductance, as well as ENaC activity, compared to air controls (Fig. 2B–D, Supplementary Fig. S1). Seven-day exposure of HBEC to PG/VG aerosols further caused a significant decrease in BK channel conductance (Fig. 2E,F, Supplementary Fig. S1). Despite causing a decrease in ENaC conductance, PG/VG aerosols resulted in a significant decrease in ciliary beating in exposed HBEC when measured after 5 days (Fig. 2G, Supplementary Fig. S1).

**Figure 3:**
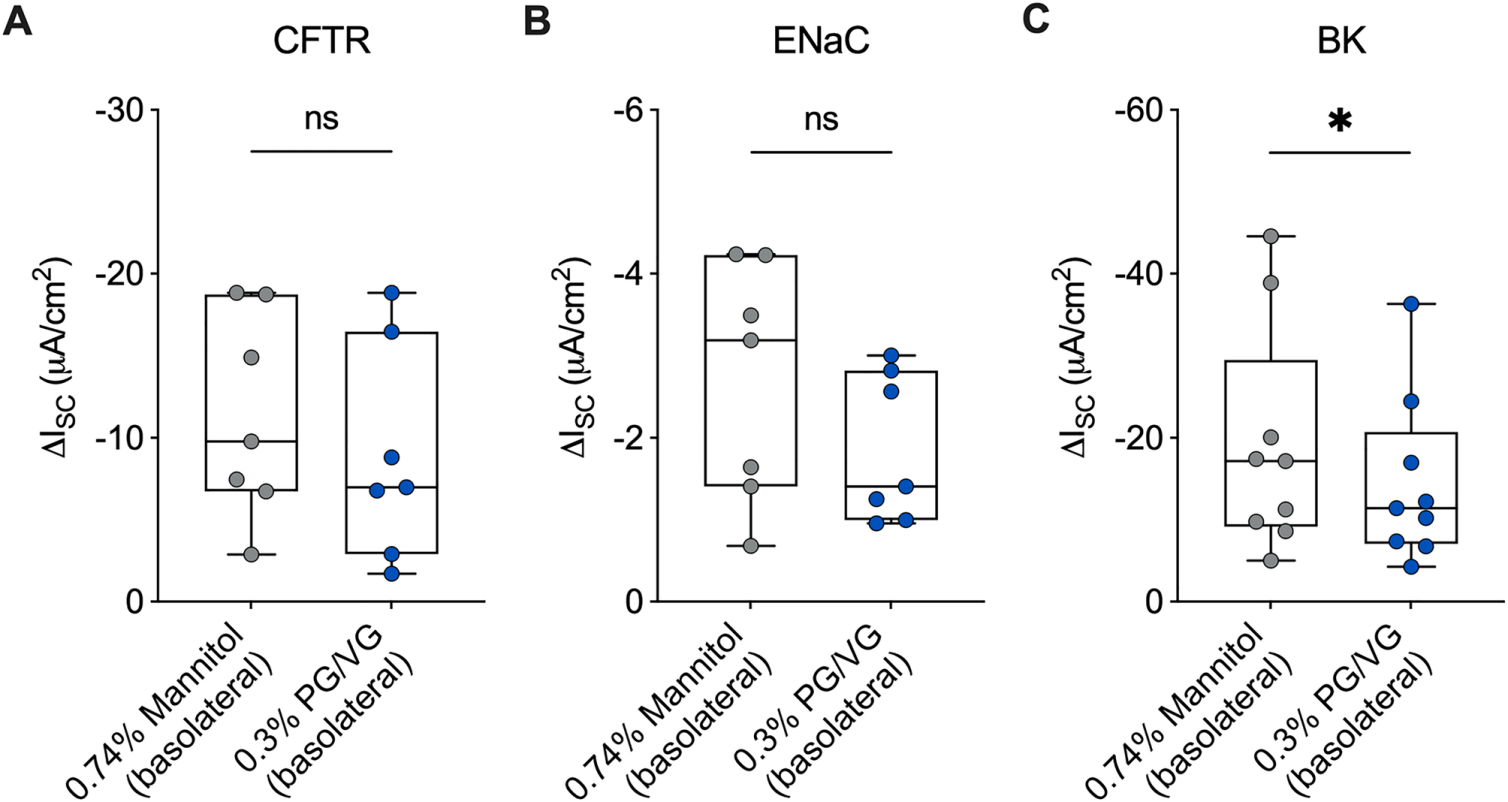
Basolateral PG/VG causes ion channel dysfunction. (**A**) CFTR conductance as measured by ∆I_*SC*_ following CFTR_inh_-172 in HBEC exposed to basolateral mannitol (0.74%) control or basolateral PG/VG (0.3%) for 24 h. n = 7 ALI cultures from 7 donors. (**B**) ENaC conductance as measured by ∆I_*SC*_ following amiloride in HBEC exposed to control or basolateral PG/VG (0.3%) for 24 h. n = 7 ALI cultures from 7 donors. (**C**) BK conductance as measured by ∆I_*SC*_ following ATP in HBEC exposed to control or basolateral PG/VG (0.3%) for 24 h. n = 9 ALI cultures from 5 donors. *Statistics:* Data shown as median (line), 25th to 75th percentiles (box), and minimum to maximum values (whiskers). *p < 0.05, Wilcoxon test.

Thermal degradation products caused by heating of PG/VG by the atomizer coil can contribute to ion channel dysfunction^16^. To test whether non-vaped PG/VG e-liquid also contributes to ion channel dysfunction, we added PG/VG e-liquid in the basolateral media of primary HBEC and assessed ion channel function after 24 h. PG/VG e-liquid was added basolaterally to avoid any confounding effects of apically added fluid on ion channel function. Basolateral PG/VG (0.3%) decreased CFTR and ENaC activities compared to an osmotic (0.74% mannitol) control, but the change was not statistically significant (Fig. 3A,B, Supplementary Fig. S2). On the other hand, basolateral PG/VG significantly decreased BK channel conductance compared to the control (Fig. 3C, Supplementary Fig. S2). Thus, non-vaped PG/VG has differential effects on ion channel function compared to vaped PG/VG in the airway epithelium.

### PG/VG aerosols induce the mRNA expression of inflammatory markers

**Figure 4:**
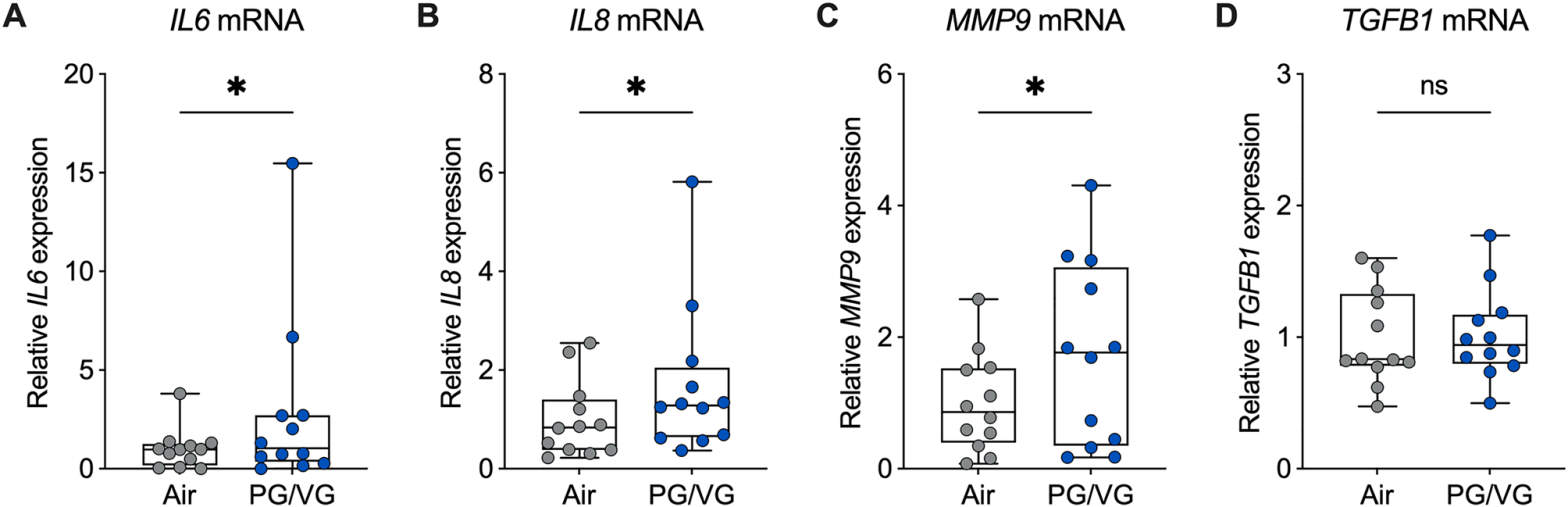
PG/VG aerosols induce the mRNA expression of inflammatory markers. (**A-D**) Relative expression levels of *IL6* (**A**), *IL8* (**B**), *MMP9* (**C**), and *TGFB1* (**D**) mRNAs in HBEC exposed to air or PG/VG aerosols for seven days. n = 12 ALI cultures from 12 donors. *Statistics:* Data shown as median (line), 25th to 75th percentiles (box), and minimum to maximum values (whiskers). *p < 0.05, Wilcoxon test.

Systemic markers of inflammation, including interleukin-6 (IL-6), interleukin-8 (IL-8), matrix metalloproteinase-9 (MMP-9), and transforming growth factor-beta1 (TGF-β1) have been found to be elevated in e-cig users who vaped nicotine-containing e-liquids^30,31^. We therefore determined whether PG/VG aerosols increase the expression of these inflammatory markers in the airway epithelium in vitro. Seven-day exposure of HBEC to e-cig aerosols of PG/VG caused a significant increase in the mRNA expressions of interleukin-6 (*IL6*), *IL8*, and matrix metalloproteinase-9 (*MMP9*) compared to air controls (Fig. 4A–C, Supplementary Fig. S3). Expression of transforming growth factor-beta1 (*TGFB1*) mRNA was statistically unchanged in HBEC exposed to PG/VG aerosols (Fig. 4D, Supplementary Fig. S3).

### PG/VG aerosols induce changes in the expression of airway cell markers

**Figure 5:**
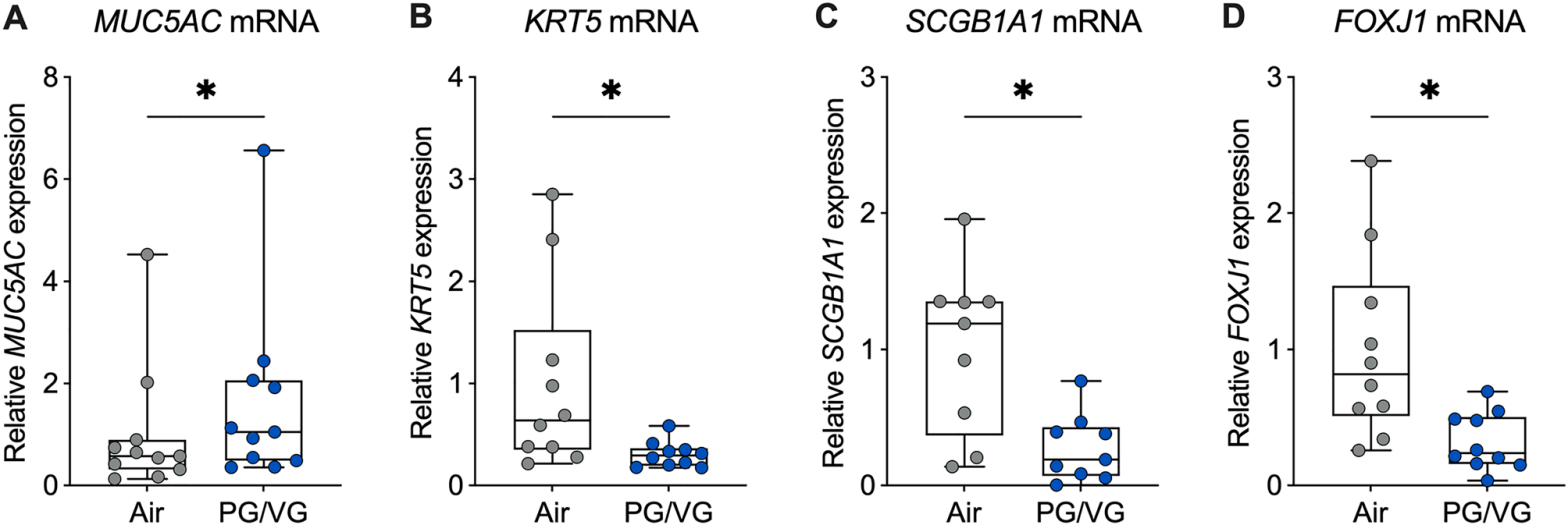
PG/VG aerosols induce changes in the expression of airway cell markers. (**A**–**D**) Relative expression levels of *MUC5AC* (n = 11 ALI cultures from 11 donors) (**A**), *KRT5* (n = 10 ALI cultures from 10 donors) (**B**), *SCGB1A1* (n = 9 ALI cultures from 9 donors) (**C**), and *FOXJ1* (n = 10 ALI cultures from 10 donors) (**D**) mRNAs in HBEC exposed to air or PG/VG aerosols for seven days. *Statistics:* Data shown as median (line), 25th to 75th percentiles (box), and minimum to maximum values (whiskers). *p < 0.05, Wilcoxon test.

To determine whether e-cig aerosols of PG/VG induce changes in the differentiation of exposed HBEC, we analyzed the mRNA expression of cell type-specific markers. HBEC exposed to PG/VG aerosols showed a significant increase in the mRNA expression of *MUC5AC*, a goblet cell marker (Fig. 5A, Supplementary Fig. S4). On the other hand, seven-day exposure of HBEC to PG/VG aerosols caused a significant decrease in the mRNA expressions of the basal cell marker keratin 5 (*KRT5*), the club cell marker secretoglobin 1A1 (*SCGB1A1*), and the ciliated cell marker forkhead box J1 (*FOXJ1*) compared to air controls (Fig. 5B–D, Supplementary Fig. S4). These data suggest that seven-day exposure of HBEC to PG/VG aerosols shifts the airway epithelium to a more mucus secreting cell population.

### PG/VG aerosols increase MUC5AC expression and decrease acetylated α-tubulin expression

**Figure 6:**
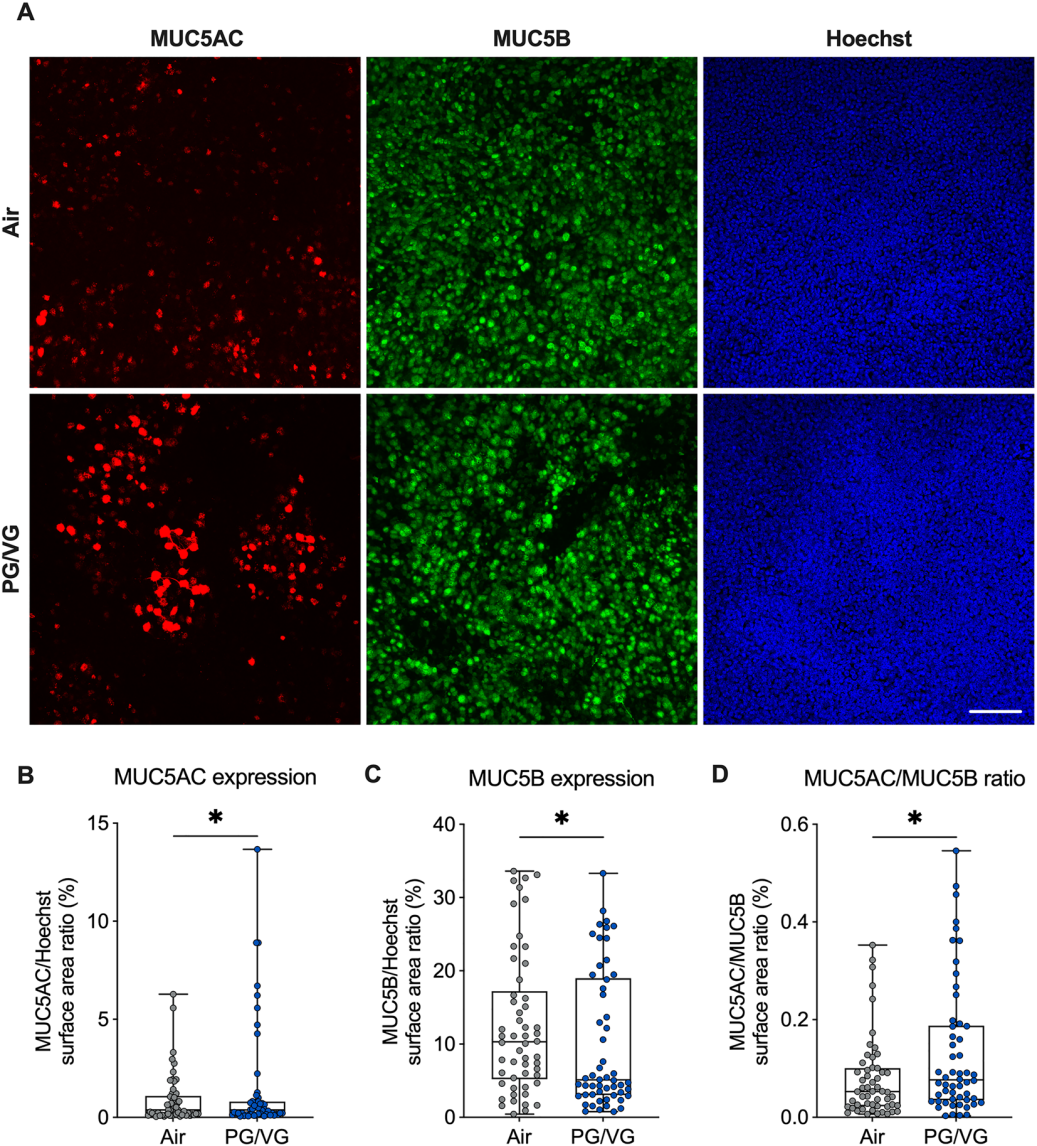
PG/VG aerosols increase MUC5AC expression. (**A**) Representative confocal image stacks of MUC5AC (red) and MUC5B (green) expression, and Hoechst stain (blue) in HBEC exposed to air or PG/VG e-cig aerosols for seven days. Scale bar, 100 µm. (**B**, **C**) Quantification of MUC5AC and MUC5B expression expressed as surface area labeling of MUC5AC/Hoechst (**B**) and MUC5B/Hoechst (**C**), respectively. (**D**) Surface area labeling of MUC5AC and MUC5B expressed as a ratio. n = 9 ALI cultures from 9 donors, which were sampled 6 times each, resulting in 54 datapoints included in the permutation analysis and shown in the figure. *Statistics:* Data shown as median (line), 25th to 75th percentiles (box), and minimum to maximum values (whiskers). *p < 0.05, LMM permutation test.

To determine whether the increase in *MUC5AC* mRNA expression correlated with increased expression of MUC5AC protein, we performed immunostaining with antibodies against MUC5AC on HBEC exposed to air or PG/VG e-cig aerosols for seven days. HBEC exposed to PG/VG aerosols showed a small but significant increase in MUC5AC expression compared to air controls (Fig. 6A,B Supplementary Fig. S5). MUC5B is the other major gel-forming mucin expressed in the airway epithelium that is required for proper mucociliary clearance^32^. PG/VG aerosols caused a significant decrease in MUC5B expression after seven days (Fig. 6A,C, Supplementary Fig. S5). The ratio of MUC5AC to MUC5B protein is associated with the severity of chronic obstructive airway diseases^33,34^. Overall, PG/VG aerosols caused a significant increase in the ratio of MUC5AC/MUC5B expression (Fig. 6D, Supplementary Fig. S5).

**Figure 7:**
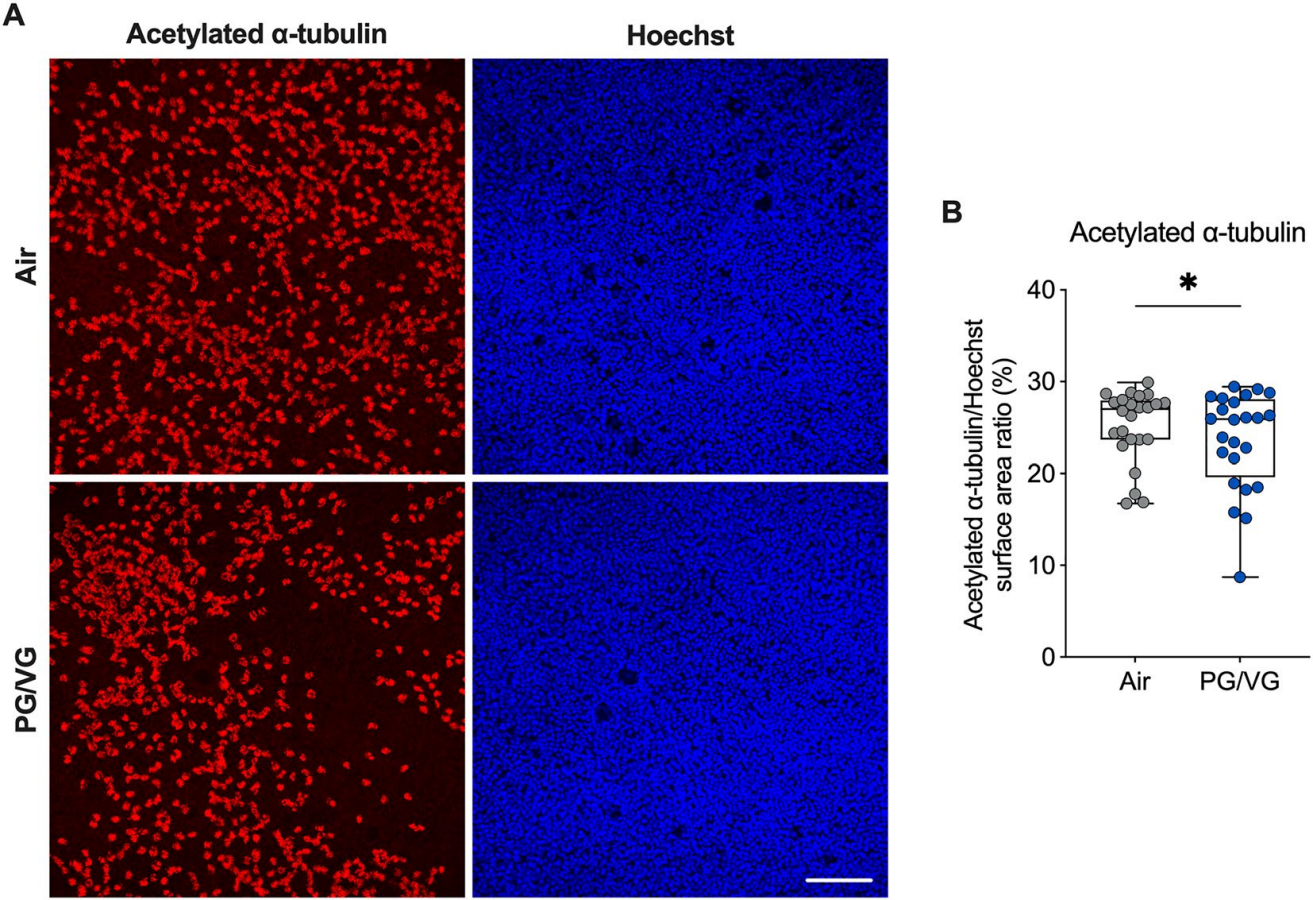
PG/VG aerosols reduce ciliation. (**A**) Representative confocal image stacks of acetylated α-tubulin (red) and Hoechst stain (blue) in HBEC exposed to air or PG/VG e-cig aerosols for seven days. Scale bar, 100 µm. (**B**) Quantification of acetylated α-tubulin expression expressed as surface area labeling of acetylated α-tubulin/Hoechst. n = 8 ALI cultures from 8 donors, which were sampled 3 times each, resulting in 24 datapoints in the permutation analysis and shown in the figure. *Statistics:* Data shown as median (line), 25th to 75th percentiles (box), and minimum to maximum values (whiskers). *p < 0.05, LMM permutation test.

We next determined whether the decrease in *FOXJ1* expression caused by PG/VG aerosols correlated with reduced ciliation by performing immunostaining with antibodies against acetylated α-tubulin. HBEC exposed to PG/VG aerosols showed a small but significant decrease in surface area staining of acetylated α-tubulin after seven days compared to air controls (Fig. 7, Supplementary Fig. S6).

### PG/VG aerosols increase mucus concentration and MMP-9 activity in sheep in vivo

**Figure 8:**
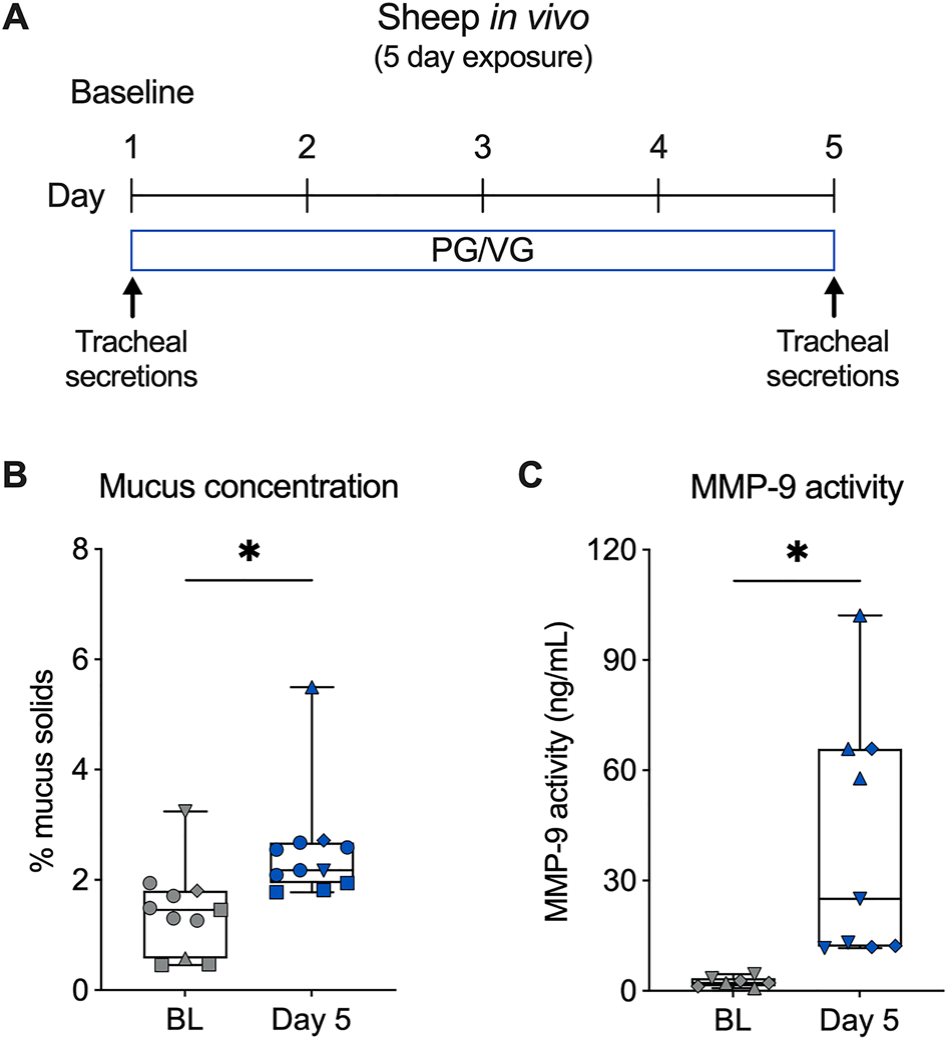
PG/VG aerosols increase mucus concentration and MMP-9 activity in sheep in vivo. (**A**) Study design for in vivo exposures of sheep to e-cig aerosols of PG/VG. (**B**) Quantification of mucus concentration (% mucus solids) measured from tracheal secretions of sheep at baseline (BL) or after five-day exposure to PG/VG e-cig aerosols. n = 5 sheep (represented by different shapes), which were sampled 1 time (3 sheep), 5 times (1 sheep), and 3 times (1 sheep), resulting in 11 datapoints in the permutation analysis and shown in the figure. (**C**) Quantification of MMP-9 activity measured from tracheal secretions of sheep at baseline (BL) or after five-day exposure to PG/VG e-cig aerosols. n = 3 sheep (represented by different shapes), which were sampled 2 times (2 sheep) and 3 times (1 sheep) for baseline and 3 times each for PG/VG, resulting in 7 (baseline) and 9 (PG/VG) datapoints in the permutation analysis and shown in the figure. *Statistics:* Data shown as median (line), 25th to 75th percentiles (box), and minimum to maximum values (whiskers). *p < 0.05, LMM permutation test.

Finally, to determine the effects of PG/VG e-cig aerosols in the airways in vivo, we used our previously established large animal (sheep) model and exposed sheep to 80 puffs per day (40 puffs per session twice daily) of PG/VG e-cig aerosols for five days (Fig. 8A). Tracheal secretions were collected at baseline and at day five after PG/VG aerosol exposure. Five-day exposure of sheep to PG/VG aerosols caused a significant increase in mucus concentration measured from tracheal secretions (Fig. 8B). Furthermore, MMP-9 activity measured from tracheal secretions was significantly elevated after five days of PG/VG aerosol exposure (Fig. 8C).

## Discussion

Although the effects of PG and/or VG in the lung are generally regarded as detrimental, different model systems and exposures have led to an unclear picture of the impact of these delivery vehicles on mucociliary clearance and airway inflammation. For example, intratracheal instillation of PG in mice for two weeks caused a significant increase in the number of inflammatory cells and lipid-laden macrophages in bronchoalveolar lavage fluid (BALF), pulmonary fibrosis, and a significant decrease in airflow during expiration^35^. However, these changes were in response to PG doses 47–137 times higher than daily PG exposure experienced by e-cig users. Instillation of VG in mice for two weeks was largely innocuous^35^. Mice exposed to e-cig aerosols of PG/VG (1:1 ratio), in the absence of nicotine and flavors, showed a significant increase in the number of macrophages and oxidative stress markers in BALF after 3 days of exposure^36^. However, these changes were not observed after 4 weeks of exposure and only observed in mice exposed to e-cig aerosols of PG/VG with nicotine and tobacco flavor^36^. Chronic exposures of mice to e-cig aerosols of PG/VG, without nicotine and flavors, further failed to induce lung inflammation^36,37^. Mice exposed to PG/VG aerosols for 4 months did show altered lipid content in the lung, disrupting surfactant homeostasis. Moreover, these mice exhibited an impaired ability to control infection and inflammation after a viral challenge^37^. Whether the effects of PG/VG in rodent models reflect the airway response to PG/VG aerosols in humans remain unclear.

Exposure of primary HBEC to high concentrations of PG/VG (3%) inhibited glucose transport, but also decreased transepithelial resistance and increased epithelial permeability^38^. 16HBE cells exposed to PG/VG also showed concentration-dependent decreases in transepithelial resistance^39^. Aerosols of PG and VG generated from a high-wattage e-cig device were also found to cause a significant increase in IL-6 and IL-8 protein levels in 16HBE cells 2-h post-exposure^40^. Although there are few clinical studies investigating the health effects of e-cig use in never-smokers and never-vapers, these studies have raised the possibility that e-cig aerosols of PG and/or VG may be sufficient to induce airway inflammation. A small clinical study investigating the pulmonary effects of vaping e-cigs containing only PG/VG found no significant difference in inflammatory cell count and cytokine expression in BALF after four weeks between e-cig users and a non-user control group^41^. However, the authors found statistically significant positive correlations between urinary PG levels and total cell concentration and lymphocyte counts, as well as changes in IL-8, IL-13, and TNF-α expression levels^41^. Our recent clinical study in never-smoker, never-vapers further revealed trending increases in the nasal expression of inflammatory markers, including IL-6, IL-8, and MMP-9, after one week of vaping sole VG-containing e-liquids^19^. Unfortunately, the small sample size of the cohorts was a limitation of the study and did not allow for statistical comparisons.

Our data here show that exposure of the airway epithelium to e-cig aerosols of PG/VG reduces ciliary beating and increases the expression of MUC5AC. The decrease in ciliary beating is likely due to dysfunction of CFTR and BK channels, both of which are critical for proper airway hydration. Reduced CFTR function leads to reduced fluid transport resulting in mucus hyperconcentration, a central feature of chronic airway diseases^42^. Similarly, BK channel dysfunction leads to reduced airway surface liquid volumes, resulting in impaired ciliary beating^24,43,44^. Recent studies are now beginning to reveal how PG and VG aerosols cause ion channel dysfunction. Heating of VG is known to produce acrolein which can disrupt CFTR function^16,45^. However, VG can also incorporate into the cell membrane to reduce membrane fluidity^19,46^. Indeed, changes to membrane fluidity have been demonstrated to alter CFTR function^47^. Our previous work also found that PG e-cig aerosols can inhibit BK function^20^. Methylglyoxal is a toxic α-dicarbonyl compound that is produced from the heating of PG by the atomizer coil^48–50^. PG can also be metabolized to methylglyoxal by alcohol dehydrogenase enzymes expressed in the airway epithelium^20^. Aside from its toxic effects, methylglyoxal can also modify arginine residues to alter protein structure and function^51^. In this case, methylglyoxal was shown to disrupt the interaction between the major pore-forming alpha subunit hSlo1 and gamma regulatory subunit LRRC26 of the BK channel to inhibit its function^20^. Our data demonstrating that non-vaped PG/VG e-liquid can cause BK dysfunction are consistent with these findings.

The observed increase in MUC5AC expression by PG/VG aerosols reported here is further consistent with a study by Ghosh et al. who demonstrated that exposure of HBEC to e-cig aerosols of PG/VG (55:45 ratio) caused a significant increase in MUC5AC expression independent of the presence of nicotine^46^. The authors further showed that MUC5AC levels in nasal epithelia from mice were significantly increased after exposure to PG/VG aerosols^46^. PG/VG aerosols also increased expression of MUC5AC, but not MUC5B, in cultures of human nasal epithelial cells^52,53^. However, it’s unclear how PG/VG aerosols induce MUC5AC expression. Our data suggest that prolonged exposure of the airway epithelium to PG/VG aerosols may lead to goblet cell hyperplasia. The increase in *MUC5AC* expression correlated with reduced expression of the basal cell marker *KRT5* as well as the club cell marker *SCGB1A1*, suggesting PG/VG aerosols possibly shift the differentiation of basal and club cells to goblet cells. PG/VG aerosols further caused a significant decrease in MUC5B expression, resulting in greater MUC5AC/MUC5B ratios. This is particularly relevant given that higher MUC5AC/MUC5B ratios are associated with severity of muco-obstructive diseases^33,34^. The overall reduction in ciliation by PG/VG aerosols further suggests a possible shift in cell types may occur after prolonged exposures.

In a recent clinical study, four patients with a 3–8 year history of e-cig use were all found to have constrictive bronchiolitis and MUC5AC overexpression in the respiratory epithelium^54^. Interestingly, one patient only vaped e-liquid containing PG and VG, without nicotine, flavors, or other substances. Although all four patients had a history of smoking and used e-cigs for cessation, studies have shown that MUC5AC concentrations in people who quit smoking are similar to concentrations in healthy non-smokers^34^. Thus, the elevation in MUC5AC levels is likely maintained by the PG/VG in the e-liquid, further suggesting that even e-cig aerosols without nicotine and flavorings can cause harm in the airways.

Our study adds to the current body of evidence suggesting that e-cig aerosols of PG/VG are not innocuous in the airway. However, our study had limitations. First, our study was not powered to detect differences based on sex as a biological variable. Second, basolateral PG/VG exposures may not replicate vaped PG/VG aerosols. Third, although our study showed significant changes in ion channel function and the expression of inflammation and airway cell markers in response to PG/VG aerosols after seven days, longer-term exposures may be necessary to mimic the effects of chronic e-cig use in humans. Finally, although the use of the permutation test on the mixed model estimates is likely valid given the assumed conditional exchangeability of the observations, our sensitivity analyses combining responses within each subject were not significant except for the MUC5AC/MUC5B ratios. This is unsurprising given that after combining results within each subject we had very small sample sizes (e.g., n = 3 sheep for MMP-9 activity) and highlights the need for further verification.

## Conclusion

Our data here demonstrate that e-cig aerosols of PG/VG likely induce mucus hyperconcentration in vivo. These changes could be caused in part by the dysfunction of ion channels important for airway hydration. We further show that PG/VG aerosols increase the expression of inflammatory markers and MUC5AC. Our data suggest that PG/VG aerosols may possibly induce goblet cell hyperplasia; however, further experimentation will be necessary to firmly establish these effects. Overall, these data suggest that long-term use of e-cigs containing PG/VG are likely to have negative consequences in the airway.

### Supplementary Information

Supplementary Figures.

Supplementary Table S1.

Supplementary Table S2.

## Data Availability

The data and materials presented here are available upon reasonable request from the corresponding author.

## Abbreviations

ALI: Air–liquid interface
BALF: Bronchoalveolar lavage fluid
BK: Large conductance, Ca^2+^-activated, and voltage-dependent K^+^
CBF: Ciliary beat frequency
CFTR: Cystic fibrosis transmembrane conductance regulator
COPD: Chronic obstructive pulmonary disease
e-cig: E-cigarette
FOXJ1: Forkhead box protein J1
HBEC: Human bronchial epithelial cells
IL-6: Interleukin-6
IL-8: Interleukin-8
KRT5: Keratin 5
MMP-9: Matrix metalloproteinase-9
MUC5AC: Mucin 5AC
MUC5B: Mucin 5B
PG: Propylene glycol
SCGB1A1: Secretoglobin family 1A member 1
TGFB1: Transforming growth factor-beta1
VG: Vegetable glycerin
