# Supplementary material for: The combination of propylene glycol and vegetable glycerin e-cigarette aerosols induces airway inflammation and mucus hyperconcentration
Source: Sci Rep. 2024 Jan 23;14:1942. doi: 10.1038/s41598-024-52317-8 (PMC10803801; doi:10.1038/s41598-024-52317-8)
Supplement: Supplementary file 1 — Supplementary Figures. [file 41598_2024_52317_MOESM1_ESM.docx]

**Supplementary Figures**

**Supplementary Figure S1**

**Supplementary Figure S1.** Sex-specific effects of PG/VG aerosols on ion channel function in HBEC *in vitro* (supplement to Figure 2). (**A**) CFTR conductance as measured by ΔI*_SC_* following CFTR_inh_-172 in HBEC exposed to air or PG/VG aerosols for seven days separated by sex. (**B**) ENaC conductance as measured by ΔI*_SC_* following amiloride in HBEC exposed to air or PG/VG aerosols for seven days separated by sex. (**C**) BK conductance as measured by ΔI*_SC_* following ATP in HBEC exposed to air or PG/VG aerosols for seven days separated by sex. (**D**) Quantification of ciliary beat frequency (CBF) from whole field analysis in HBEC exposed to air or PG/VG aerosols for five days separated by sex. Data shown as median (line), 25^th^ to 75^th^ percentiles (box), and minimum to maximum values (whiskers).

**Supplementary Figure S2**

**Supplementary Figure S2.** Sex-specific effects of basolateral PG/VG on ion channel function in HBEC *in vitro* (supplement to Figure 3). (**A**) CFTR conductance as measured by ΔI*_SC_* following CFTR_inh_-172 in HBEC exposed to basolateral mannitol (0.74%) control or basolateral PG/VG (0.3%) for 24 hours separated by sex. (**B**) ENaC conductance as measured by ΔI*_SC_* following amiloride in HBEC exposed to basolateral mannitol (0.74%) control or basolateral PG/VG (0.3%) for 24 hours separated by sex. (**C**) BK conductance as measured by ΔI*_SC_* following ATP in HBEC exposed to basolateral mannitol (0.74%) control or basolateral PG/VG (0.3%) for 24 hours separated by sex. Data shown as median (line), 25^th^ to 75^th^ percentiles (box), and minimum to maximum values (whiskers).

**Supplementary Figure S3**

**Supplementary Figure S3.** Sex-specific effects of PG/VG aerosols on the mRNA expression of inflammatory markers in HBEC *in vitro* (supplement to Figure 4). (**A-D**) Relative expression levels of *IL6* (**A**), *IL8* (**B**), *MMP9* (**C**), and *TGFB1* (**D**) mRNAs in HBEC exposed to air or PG/VG aerosols for seven days separated by sex. Data shown as median (line), 25^th^ to 75^th^ percentiles (box), and minimum to maximum values (whiskers).

**Supplementary Figure S4**

**Supplementary Figure S4.** Sex-specific effects of PG/VG aerosols on the expression of airway cell markers in HBEC *in vitro* (supplement to Figure 5). (**A-D**) Relative expression levels of *MUC5AC* (**A**), *KRT5* (**B**), *SCGB1A1* (**C**), and *FOXJ1* (**D**) mRNAs in HBEC exposed to air or PG/VG aerosols for seven days separated by sex. Data shown as median (line), 25^th^ to 75^th^ percentiles (box), and minimum to maximum values (whiskers).

**Supplementary Figure S5**

**Supplementary Figure S5.** Sex-specific effects of PG/VG aerosols on MUC5AC and MUC5B expression in HBEC *in vitro* (supplement to Figure 6). (**A,B**) Quantification of MUC5AC and MUC5B expression expressed as surface area labeling of MUC5AC/Hoechst (**A**) and MUC5B/Hoechst (**B**) separated by sex. (**C**) Surface area labeling of MUC5AC and MUC5B expressed as a ratio separated by sex. Data shown as median (line), 25^th^ to 75^th^ percentiles (box), and minimum to maximum values (whiskers).

**Supplementary Figure S6**

**Supplementary Figure S6.** Sex-specific effects of PG/VG aerosols on ciliation in HBEC *in vitro* (supplement to Figure 7). Quantification of acetylated α-tubulin expression expressed as surface area labeling of acetylated α-tubulin/Hoechst separated by sex. Data shown as median (line), 25^th^ to 75^th^ percentiles (box), and minimum to maximum values (whiskers).
