# Supplementary material for: The combination of propylene glycol and vegetable glycerin e-cigarette aerosols induces airway inflammation and mucus hyperconcentration
Source: Sci Rep. 2024 Jan 23;14:1942. doi: 10.1038/s41598-024-52317-8 (PMC10803801; doi:10.1038/s41598-024-52317-8)
Supplement: Supplementary file 2 — Supplementary Table S1. [file 41598_2024_52317_MOESM2_ESM.docx]

**Supplementary Table S1**

**Characteristics of never-smoker donor lungs used in this study.**

| **Age (yrs)** | **Gender** | **Race/Ethnicity** | **Cause of Death** |
| --- | --- | --- | --- |
| 15 | Female | Black | Drug overdose |
| 17 | Female | White | Motor vehicle accident |
| 19 | Female | White | Motor vehicle accident |
| 19 | Female | Hispanic | Unknown |
| 19 | Female | White | Unknown |
| 20 | Female | White | Drowning |
| 20 | Female | White | Head trauma |
| 23 | Female | White | Head trauma |
| 27 | Female | Hispanic | Motor vehicle accident |
| 29 | Female | White | Anoxia |
| 33 | Female | White | Motor vehicle accident |
| 38 | Female | White | Unknown |
| 49 | Female | White | Head trauma |
| 50 | Female | White | Anoxia |
| 53 | Female | White | Unknown |
| 60 | Female | White | Unknown |
| 18 | Male | White | Unknown |
| 18 | Male | White | Motor vehicle accident |
| 20 | Male | Unknown | Gunshot wound |
| 21 | Male | White | Anoxia |
| 21 | Male | Unknown | Motor vehicle accident |
| 22 | Male | Hispanic | Gunshot wound |
| 23 | Male | White | Unknown |
| 24 | Male | Black | Unknown |
| 32 | Male | Black | Gunshot wound |
| 36 | Male | Hispanic | Unknown |
| 40 | Male | White | Unknown |
| 46 | Male | White | Intracerebral hemorrhage |
| 55 | Male | White | Cardiac arrest |
| 67 | Male | White | Anoxia |
| 79 | Male | White | Intracerebral hemorrhage |
