# Supplementary material for: The combination of propylene glycol and vegetable glycerin e-cigarette aerosols induces airway inflammation and mucus hyperconcentration
Source: Sci Rep. 2024 Jan 23;14:1942. doi: 10.1038/s41598-024-52317-8 (PMC10803801; doi:10.1038/s41598-024-52317-8)
Supplement: Supplementary file 3 — Supplementary Table S2. [file 41598_2024_52317_MOESM3_ESM.docx]

**Supplementary Table S2**

**Comparisons of p values for LMM permutation test vs. Paired permutation t-test.**

| **Figure** | **Measurement** | **LMM permutation test**  (p value) | **Paired permutation t-test**  (p value) |
| --- | --- | --- | --- |
| 2G | CBF | 0.000012 | 0.25 |
| 6A | MUC5AC/Hoechst | 0.026 | 0.375 |
| 6B | MUC5B/Hoechst | 0.017 | 0.172 |
| 6C | MUC5AC/MUC5B | 0.002 | 0.007 |
| 7B | Tubulin/Hoechst | 0.047 | 0.086 |
| 8B | Mucus solids | 0.004 | 0.16 |
| 8C | MMP-9 activity | 0.0005 | 0.25 |
